# A DREAM Challenge to Build Prediction Models for Short-Term Discontinuation of Docetaxel in Metastatic Castration-Resistant Prostate Cancer

**DOI:** 10.1200/CCI.17.00018

**Published:** 2017-08-04

**Authors:** Fatemeh Seyednasrollah, Devin C. Koestler, Tao Wang, Stephen R. Piccolo, Roberto Vega, Russell Greiner, Christiane Fuchs, Eyal Gofer, Luke Kumar, Russell D. Wolfinger, Kimberly Kanigel Winner, Chris Bare, Elias Chaibub Neto, Thomas Yu, Liji Shen, Kald Abdallah, Thea Norman, Gustavo Stolovitzky, Howard R. Soule, Christopher J. Sweeney, Charles J. Ryan, Howard I. Scher, Oliver Sartor, Laura L. Elo, Fang Liz Zhou, Justin Guinney, James C. Costello, Prostate Cancer DREAM Challenge Community

**Affiliations:** **Fatemeh Seyednasrollah** and **Laura L. Elo**, Turku Centre for Biotechnology; University of Turku; Åbo Akademi University, Turku, Finland; **Devin C. Koestler**, University of Kansas Medical Center, Kansas City, KS; **Tao Wang**, University of Texas Southwestern Medical Center, Dallas, TX; **Stephen R. Piccolo**, Brigham Young University, Provo; University of Utah, Salt Lake City, Utah, UT; **Roberto Vega**, **Russell Greiner**, and **Luke Kumar**, University of Alberta; Alberta Innovates Centre for Machine Learning, Edmonton, Alberta, Canada; **Christiane Fuchs**, Helmholtz Zentrum München, Neuherberg; Technische Universität München, Garching, Germany; **Eyal Gofer**, The Hebrew University, Jerusalem, Israel; **Russell D. Wolfinger**, SAS Institute, Cary, NC; **Kimberly Kanigel Winner** and **James C. Costello**, University of Colorado, Anschutz Medical Campus, Aurora, CO; **Chris Bare**, **Elias Chaibub Neto**, **Thomas Yu**, **Thea Norman**, and **Justin Guinney**, Sage Bionetworks, Seattle, WA; **Liji Shen** and **Fang Liz Zhou**, Sanofi, Bridgewater, NJ; **Kald Abdallah**, AstraZeneca, Gaithersburg, MD; **Gustavo Stolovitzky**, IBM Research, Yorktown Heights; **Howard I. Scher**, Memorial Sloan Kettering Cancer Center and Weill Cornell Medical College, New York, NY; **Howard R. Soule**, Prostate Cancer Foundation, Santa Monica; **Charles J. Ryan**, University of California, San Francisco, CA; **Christopher J. Sweeney**, Dana-Farber Cancer Institute and Brigham and Women's Hospital, Harvard Medical School, Boston, MA; and **Oliver Sartor**, Tulane University, New Orleans, LA.

## Abstract

**Purpose:**

Docetaxel has a demonstrated survival benefit for patients with metastatic castration-resistant prostate cancer (mCRPC); however, 10% to 20% of patients discontinue docetaxel prematurely because of toxicity-induced adverse events, and the management of risk factors for toxicity remains a challenge.

**Patients and Methods:**

The comparator arms of four phase III clinical trials in first-line mCRPC were collected, annotated, and compiled, with a total of 2,070 patients. Early discontinuation was defined as treatment stoppage within 3 months as a result of adverse treatment effects; 10% of patients discontinued treatment. We designed an open-data, crowd-sourced DREAM Challenge for developing models with which to predict early discontinuation of docetaxel treatment. Clinical features for all four trials and outcomes for three of the four trials were made publicly available, with the outcomes of the fourth trial held back for unbiased model evaluation. Challenge participants from around the world trained models and submitted their predictions. Area under the precision-recall curve was the primary metric used for performance assessment.

**Results:**

In total, 34 separate teams submitted predictions. Seven models with statistically similar area under precision-recall curves (Bayes factor ≤ 3) outperformed all other models. A postchallenge analysis of risk prediction using these seven models revealed three patient subgroups: high risk, low risk, or discordant risk. Early discontinuation events were two times higher in the high-risk subgroup compared with the low-risk subgroup. Simulation studies demonstrated that use of patient discontinuation prediction models could reduce patient enrollment in clinical trials without the loss of statistical power.

**Conclusion:**

This work represents a successful collaboration between 34 international teams that leveraged open clinical trial data. Our results demonstrate that routinely collected clinical features can be used to identify patients with mCRPC who are likely to discontinue treatment because of adverse events and establishes a robust benchmark with implications for clinical trial design.

## INTRODUCTION

Despite decades of research and advances in treatment, the long-term prognosis of metastatic castration-resistant prostate cancer (mCRPC) remains poor.^1^ Docetaxel was the first cytotoxic drug to improve survival and quality of life in patients with mCRPC^2,3^ and has remained a standard first-line chemotherapy for the treatment of mCRPC. Although several clinical trials have confirmed the population-level survival and palliative benefits of docetaxel,^4,5^ a significant fraction of patients do not respond to docetaxel, and within approximately 8 months, nearly all patients become resistant to treatment or stop therapy.^2,3^ Of patients who initially experience a response to docetaxel, 10% to 20% prematurely discontinue as a result of toxicity-induced adverse events (AEs) that include anemia, (febrile) neutropenia, fatigue, GI complications, and neuropathies.^6-8^ Managing the risk factors for toxicity is a major challenge, as they may diminish a patient’s quality of life without extending it.

As docetaxel-based chemotherapy continues to play an important role in the treatment of mCRPC and, more recently, hormone-sensitive metastatic prostate cancer,^9^ it is important to prospectively identify patients for whom a docetaxel-based regimen is likely to be poorly tolerated and to result in AE and potentially early treatment failure. In particular, such knowledge could be used to identify patients for pre-emptive clinical interventions and/or supportive care before chemotherapy or to direct patients to alternative treatment regimens. In addition, establishing quantitative benchmarks for identifying patients who are at high risk for early treatment discontinuation can be used to facilitate more efficient clinical trial design. Prognostic models to predict overall patient survival in mCRPC have been previously described^10-14^; however, whether early treatment discontinuation as a result of adverse events can be predicted on the basis of a patient’s baseline clinical characteristics remains an unanswered question. Within the clinical trial data used in this study, approximately 10% of patients with mCRPC discontinued treatment within 3 months of starting docetaxel. Given this low percentage of patients, access to a sufficiently powered data set is a major factor in being able to address the question of whether treatment discontinuation can be predicted.

Here, we report the results from the Prostate Cancer DREAM (Dialogue for Reverse Engineering Assessment and Methodology) Challenge, the first crowd-sourced competition in mCRPC. The aim of this challenge was to determine whether baseline clinical characteristics can be used to predict patients who will discontinue their docetaxel-based treatment because of adverse events. This challenge builds on the open clinical trial data initiative of Project Data Sphere, LLC, a nonprofit initiative of the CEO Roundtable on Cancer’s Life Consortium. The comparator arms of four phase III clinical trials with a total of 2,070 patients were collected, cleaned, annotated, and made public, removing the privacy and legal barriers for open data access. During a 5-month period, 34 teams from around the world worked independently to address the challenge, which resulted in novel models for the prediction of discontinuation and identification of clinical variables that are associated with treatment discontinuation. We also demonstrate how clinical trial design can be optimized through the use of these models. Finally, we present a new paradigm for addressing challenges in biomedical clinical informatics through a postchallenge, community-based collaboration between challenge organizers and participating teams to evaluate and refine risk prediction models.

## PATIENTS AND METHODS

### Trial Selection, Patient Population, and Data Processing

Data used in this challenge were compiled on the basis of provider-deidentified comparator arms of four phase III prostate cancer clinical trials (ASCENT2^15^: n = 476, 105 patients discontinued docetaxel within 3 months as a result of AE or possible AE; VENICE^16^: n = 598, 51 discontinued patients; MAILSAIL^17^: n = 526, 41 discontinued patients; and ENTHUSE 33^18^: n = 470, 49 discontinued patients). All trials were randomized and shared similar inclusion and exclusion criteria. Eligible patients included those with progressive mCRPC, no previous chemotherapy, and an Eastern Cooperative Oncology Group (ECOG) performance status of 0 to 2. Detailed inclusion and exclusion criteria of each trial can be found in Guinney et al^10^ and the Data Supplement. In total, data used in this challenge consisted of 2,070 patients with first-line mCRPC who were treated with a docetaxel-based treatment regimen. A total of 129 baseline clinical variables were compiled for each trial with details of data curation provided in the Data Supplement.

### Patient Discontinuation

The outcome variable—treatment discontinuation—was derived from two factors: reason for treatment discontinuation (Data Supplement) and the time from treatment initiation to discontinuation. Discontinuation of treatment was evaluated for the first 3 months of treatment or the first four cycles (12 weeks) of treatment in a 10-cycle regimen (3 weeks per cycle). Patients were labeled as discontinued if, and only if, they discontinued treatment as a result of AE or possible AE within 3 months (91.5 days) after beginning treatment. The number and percentage of patients who were assigned to a detailed list of categories are provided in the Data Supplement.

### Challenge Design, Scoring, and Evaluation

The challenge was hosted and managed on the free, cloud-based Synapse platform.^19^

ASCENT2, VENICE, and MAINSAIL data sets—clinical features and outcome—were combined to create the training data set (n = 1,600). The outcome variable for the ENTHUSE 33 data (n = 470) was withheld and used as an independent validation set to evaluate model prediction performance. Teams were tasked with developing models to predict early discontinuation of docetaxel as a result of AE or possible AE (Data Supplement). A team’s prediction was a ranked list of risk scores for all patients in the ENTHUSE 33 data; teams were allowed two submissions. Risk scores that were submitted by each team were evaluated and ranked by using the area under the precision-recall curve (AUPRC).^20^ (NOTE. Precision = positive predictive value and recall = sensitivity.) AUPRC was selected over the area under the receiver operating characteristic curve (AUROC) to take into account the highly skewed distribution of classes (10% to 20% of patients discontinuing treatment). AUPRC, unlike AUROC, emphasizes the ability of a model to predict patients who discontinue treatment (true positives) and is recommended for imbalanced data.^20-22^

The following criteria were used to determine the top teams and models: prediction performance was significantly better than a random prediction model,^23^ and performance was statistically indistinguishable compared with the model that achieved the highest AUPRC score. One-sided *P* values were computed as the probability of observing an AUPRC under the null distribution that was at least as large as the AUPRC obtained for a given team, then corrected for multiple hypothesis testing.^24^ To assess whether consecutively ranked models were distinguishable in terms of their AUPRC score, the Bayes factor^25,26^ was computed between each model and the top-ranked model. Submissions with a Bayes factor of < 3 were determined to be statistically indistinguishable as suggested by Kass and Raftery.^26^ The Bayes factor method generates a bootstrapped performance distribution between two models, where a Bayes factor of 3, for example, means that the first method outperformed the second method at a 3-to-1 ratio. Additional details can be found in the Data Supplement.

Risk scores submitted by each team were also subjected to a cumulative lift chart analysis (Data Supplement). For each team and model, we summarized the results by computing the area under the lift ratio curve and the lift ratio among patients with the highest predicted risk of early treatment discontinuation (top 5%, 10%, and 20%).

### Postchallenge Community Collaboration to Improve Patient Risk Predictions

After the completion of the challenge, hierarchical clustering was performed over the ranked patient risk scores to find a consensus pattern among the top-performing teams to identify patients who were at high or low risk of developing AEs. Unsupervised hierarchical clustering was performed by using the Manhattan distance and Ward agglomerative clustering. In addition to concordant high- and low-risk patients, we found a group of patients without any consensus risk stratification across the models, which is referred to as the discordant group. The elbow method was used to determine the number of clusters for patients; we calculated the within-group variation for different numbers of clusters, ranging from 1 to 10. The optimal number of clusters was determined at the point where the variation begins to flatten, arriving at three clusters.

To improve patient risk predictions, an ensemble-based prediction model^27^ was generated as the weighted average of the top seven models (Data Supplement). To calculate a team’s weight, each team trained their models on 70% of randomly sampled patients from the ASCENT2, VENICE, and MAINSAIL trials, then predicted risk scores for the remaining 30% of patients. This team performance established team weights. Finally, this ensemble approach was applied to the ENTHUSE 33 data and compared with individual model performances.

### Clinical Trial Model Simulations

A simulation study was conducted to quantify the benefit of incorporating patient risk for early treatment discontinuation into patient selection in terms of the sample size requirements for clinical trials (Data Supplement). We assumed a balanced two-arm randomized controlled trial—1-to-1 random assignment between treatment and control arms—and survival time as the end point. We used data from the ENTHUSE 33 trial to inform simulation parameters, then simulated 100 independent data sets in 10,000 patients using the survsim package in R.^28^ These data were used to estimate the sample size that was required to detect a survival difference (hazard ratio [HR], 1.3, 1.4, …, 2.0) between the groups at 80% statistical power and a false positive rate of 5%. Patients who were identified as being at risk for early discontinuation were excluded from random assignment, assuming different accuracies (0%, 25%, 50%, 75%, and 100%) of the baseline prediction models at identifying true cases of early discontinuation.

### Data and Method Availability

Clinical trial data can be accessed at Project Data Sphere, LLC.^29^ Method write-ups, code, and predictions for all teams are reported in the Data Supplement. Challenge documentation, overall results, scoring scripts, and data dictionary can be found at Synapse.^30^

## RESULTS

**Fig 1.**
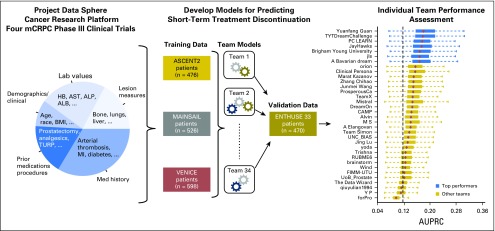
Study design. Data were acquired from Project Data Sphere Cancer Research Platform and centrally curated by the organizing team to create a standardized data set across the four studies. Three of the studies (ASCENT2, VENICE, and MAINSAIL) were selected as training sets, and a fourth data set (ENTHUSE 33) was withheld as a validation set. Teams submitted risk scores for evaluation in the validation set, which were scored and ranked by using the area under the precision-recall curve (AUPRC). The vertical dashed line in the rightmost panel represents the bootstrap estimate of the fraction of discontinuation events in the ENTHUSE33 dataset. (NOTE. Precision = positive predictive value, recall = sensitivity.) ALB, albumin; ALP, alkaline phosphatase; BMI, body mass index; HB, hemoglobin; mCRPC, metastatic castration-resistant prostate cancer; MI, myocardial infarction; TURP, transurethral resection of the prostate.

**Fig 2.**
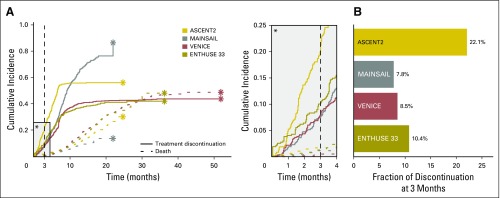
Rate and frequency of treatment discontinuation across trials. (A) Trial-specific cumulative incidence functions for treatment discontinuation as a result of adverse events or possible adverse events (solid lines) and death (dotted lines). (B) Fraction of patients with metastatic castration-resistant prostate cancer who discontinued treatment ≤ 3 months after initiation because of adverse events or possible adverse events.

The overall challenge design is illustrated in Fig 1. Across all trials, a total of 129 baseline clinical variables were made available. Although the majority of baseline clinical variables were consistent across the four trials, notable differences in the distribution of binary clinical features—primarily lesion sites—were observed (Table 1 and Data Supplement). The frequency of early discontinuation events was similar between training and validation sets (12% *v* 10% of patients, respectively), but varied across individual trials (Fig 2).

**Table 1.**
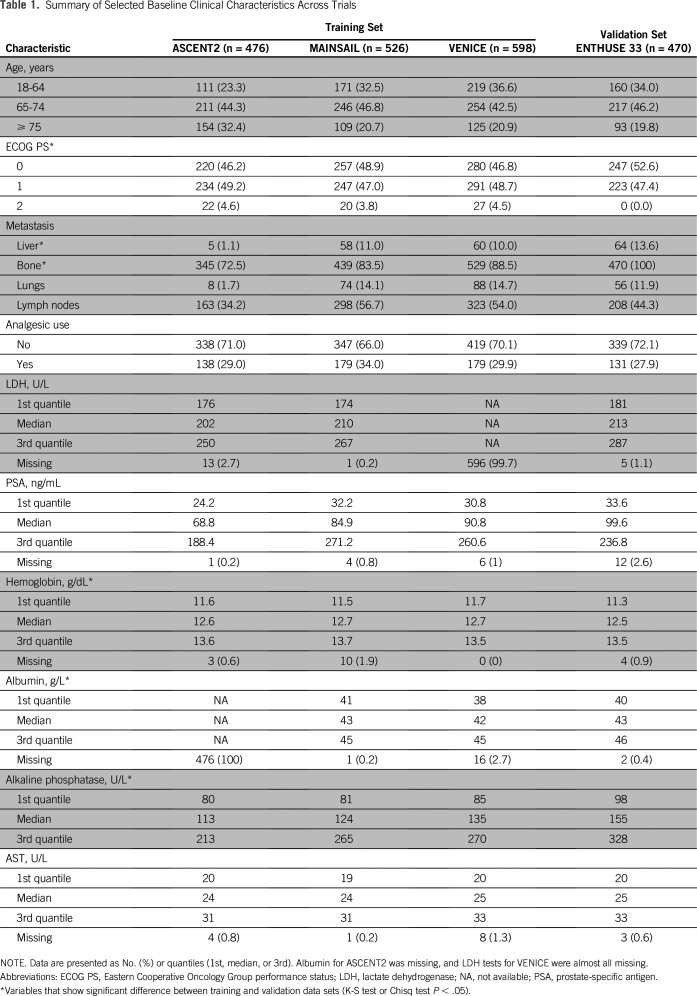
Summary of Selected Baseline Clinical Characteristics Across Trials

**Fig 3.**
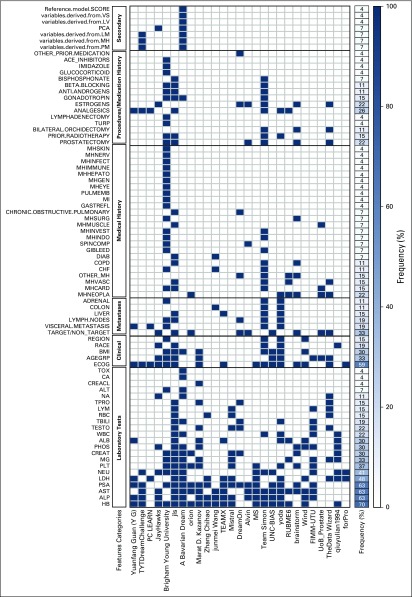
Most frequent clinical features used across all prediction models. Abbreviated terms are provided in the Data Supplement.

A total of 34 independent, international teams made 61 submissions to the challenge. A summary of each team’s approach is provided in the Data Supplement. Among teams that responded to a postchallenge survey, the five most common clinical features used in prediction models were hemoglobin (HB), alkaline phosphatase (ALP), AST, prostate-specific antigen (PSA), and ECOG performance status (Fig 3).

For evaluating team performance on the task of predicting patient discontinuation using imbalanced data (10% to 20% discontinue rate), we chose AUPRC (additional details provided in Patients and Methods and the Data Supplement). Across all submissions, AUPRC ranged between 0.088 and 0.178 (AUROC ranged between 0.55 and 0.60), with 0.104 representing the expected AUPRC for a random prediction model, which is reflective of the 10% rate of discontinuation observed in the ENTHUSE 33 trial (Fig 2 and Data Supplement). Of the 34 teams, 30 performed better than random, with seven teams performing significantly better than a random model (adjusted *P* < .10). In rank order, teams Yuanfang Guan, TYTDreamChallenge, PC LEARN, JayHawks, Brigham Young University, jls, and A Bavarian Dream achieved AUPRCs that were within a Bayes factor of 3 (Data Supplement); thus, these seven teams were identified as the challenge top performers.

A cumulative lift chart analysis was performed on each submission to provide context for their associated risk predictions. Across the top seven models, the average measure of 1.34 represents a 34% improvement in predicting short-term discontinuation compared with no risk predictions being made (Data Supplement). Restricting the above analysis to the top 10% of patients with the highest predicted risk revealed that models improved the identification of early discontinuation events by a factor of two, on average, when compared with no risk predictions being made (Data Supplement).

**Fig 4.**
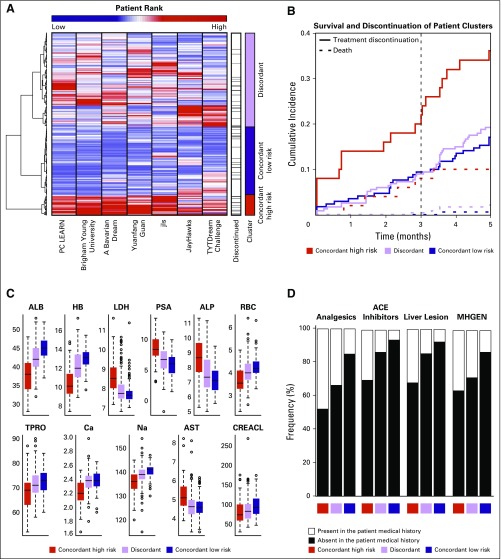
Meta-analysis of risk scores computed by the seven top-performing teams. (A) Hierarchical clustering heatmap of patients in the ENTHUSE 33 validation data set (n = 470) on the basis of their normalized ranked risk score, computed across the seven top-performing teams. (B) Kaplan-Meier curves, stratified by event type—that is, death or treatment discontinuation—across the three identified patient subgroups. (C) Distribution of baseline laboratory variables found to be significantly different between the three patient subgroups. (D) Distribution of baseline prior medical and medication variables found to be significantly different between the three patient subgroups. ACE, angiotensin-converting enzyme; ALB, albumin; ALP, alkaline phosphatase; Ca, calcium; CREACL, creatinine clearance; HB, hemoglobin; LDH, lactate dehydrogenase; MHGEN, medical history: general disorders and administration site conditions; Na, sodium; PSA, prostate-specific antigen; TPRO, total protein.

To compare the risk predictions generated by the top seven performers, we hierarchically clustered the ranked patient risk scores, which resulted in three groups of patients: patients who were consistently predicted to be at high risk of early discontinuation (concordant high risk; n = 50), patients with a consistent low risk of early discontinuation (concordant low risk; n = 170), and patients with discordant risk scores (discordant risk; n = 234 patients; Fig 4A). A nearly two-fold increase in cumulative incidence of early discontinuation was observed when the high-risk group was compared with the low-risk and discordant groups (Fig 4B). At 3 months post-treatment, 26% of patients in the concordant high-risk group discontinued docetaxel compared with only 9% in the low-risk and discordant groups. The competing risk—that is, death—was considerably elevated in the concordant high-risk group compared with the low-risk and discordant groups.

A comparison of baseline characteristics across the three groups revealed 11 statistically significant laboratory values (adjusted *P* < .05), including albumin, HB, lactate dehydrogenase, PSA, sodium, RBC, ALP, calcium, AST, creatinine clearance, and total protein (Fig 4C). In addition, ECOG performance status and metastatic liver lesions differed significantly between the concordant high-risk and low-risk groups (adjusted *P* < .05). Use of analgesics and angiotensin-converting enzyme inhibitors was significantly elevated among patients in the high-risk group (48% and 30%, respectively) compared with those in the low-risk group (15% and 5%, respectively; Fig 4D). A similar trend was observed in the frequency of patients with liver metastasis; liver lesions were reported for only 8% of patients in the concordant low-risk group compared with 32% in the high-risk group.

**Fig 5.**
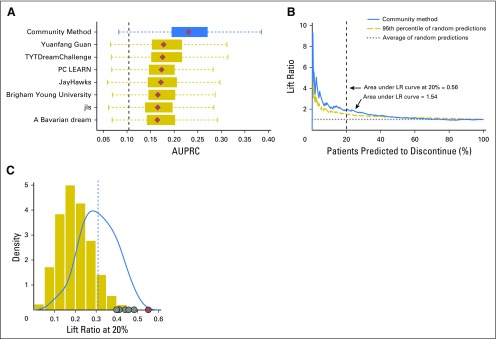
Performance of the postchallenge ensemble-based prediction model. (A) Area under the precision-recall curve (AUPRC) computed within the ENTHUSE 33 data set for the ensemble-based prediction model, along with the models developed by the seven top-performing teams. Diamonds represent the observed AUPRCs, and horizontal boxplots reflect the empirical distribution of a model’s AUPRC on the basis of 5,000 bootstrap samples generated from each models’ predictions. The vertical dotted line represents the mean AUPRC computed from 5,000 bootstrap samples generated from a random prediction model. (B) Lift ratio (LR) curve for the ensemble-based prediction model, with gray lines representing the LR curves generated for 100 random prediction models. (C) Distribution of the area under the LR curve at 20% on the basis of random prediction models (gold), all challenge submissions teams (blue), the top-performing teams (gray points), and the postchallenge ensemble-based classifier (red).

Results from previous DREAM Challenges have demonstrated that integrating predictions from multiple top-performing teams produces robust and often better results than the top individual teams.^31-33^ Motivated by these previous results and the modest correlation of risk scores across the top performers (Data Supplement), we developed a weighted average ensemble prediction model using the top seven models, with weights empirically determined (Data Supplement). Application of the ensemble-based model to the ENTHUSE 33 trial resulted in an AUPRC of 0.230 (AUROC, 0.599; Fig 5A). The ensemble-based model outperformed the top individual performers the majority of times (73% to 95% across the top seven models), and achieved a Bayes factor > 3 compared with all but one challenge submission (team Yuanfang Guan, Bayes factor, 2.75; Data Supplement). A cumulative lift chart analysis of risk predictions that were computed from the ensemble-based model demonstrated a 14% improvement over the top challenge submission (Fig 5B). Additional analysis revealed a statistically significant increase in the area under the lift ratio curve at 20% (*P* < .01; Fig 5C).

To evaluate our ensemble-based prediction model within the broader context of clinical trial design, we conducted a simulation study to compare the sample size requirements of trials that incorporate risk estimates for early treatment discontinuation as a patient inclusion criterion for the treatment arm across a range of risk prediction accuracies (Data Supplement). Results demonstrated that when patient selection considered the risk of early treatment discontinuation, fewer patients were required for the trial without a loss of statistical power. For example, if patient discontinuation was not considered, an average of 1,548 patients was needed to detect an HR of 1.30 at 80% statistical power and a false-positive rate of 5%; however, when selection into the trial was based on the ensemble-based model, the estimated sample size that was required for detecting an HR of 1.30 was reduced to 1,306 patients (Data Supplement).

## DISCUSSION

A growing number of studies support the clinical value of prediction models for early treatment discontinuation on the basis of a patient’s clinical characteristics.^34-37^ Our results show that clinical features can be used to identify patients with mCRPC who may respond adversely to docetaxel treatment. Previous prognostic models have focused on overall survival and identified important risk factors, including ALP, HB, albumin, PSA, lactate dehydrogenase, ECOG performance status, lesion site, and use of analgesics.^10,11^ By using the results from the top seven teams in this study, we confirmed that these variables are predictive of poor prognosis and also discovered several predictive variables that are related to early discontinuation of docetaxel treatment, including PSA, RBC, calcium, AST, creatinine clearance, and total protein (Fig 4). Of interest, AST was used in many of the top-performing models and was found to be significantly elevated in the high-risk versus low-risk groups for discontinuation, which is concordant with previous observations in patients who were treated with first-line chemotherapeutics in non–small-cell lung cancer.^37^ Whereas additional investigations are needed to understand the clinical and biologic implications of risk factors in predicting docetaxel-related AEs, our results present the first findings, to our knowledge, focused on toxicity-induced treatment discontinuation as a result of docetaxel in the treatment of mCRPC.

Although the performance of the challenge and postchallenge models remained modest and lacked the accuracy needed for immediate clinical application,^38^ this study is nevertheless a critical first step in the development of viable clinical tools. In particular, the challenge served to initiate the postchallenge community effort, which led to an ensemble-based prediction model that recorded performances greater than any individual model, demonstrating the power of collaborative communities as a paradigm for clinical informatics research.^39,40^ The results presented here are the product of 34 independent, international teams that were focused on addressing a common, well-defined question within a short period of time.

Of importance, this challenge is the first to establish a performance benchmark for evaluating models that predict early failure of docetaxel treatment in patients with mCRPC, which will serve as a basis for developing future prediction models. Our results are encouraging and in line with a growing emphasis on the need for innovative approaches for clinical trial design.^41^ Indeed, we demonstrated through a simulation study that identifying patients who discontinue treatment could reduce patient enrollment in clinical trials by significant numbers, especially when the desired effect size between controls and treatment is small.

We recognize that since the completion of the four trials used here, several promising therapies have emerged that have reshaped the treatment of mCRPC.^42^ The predictive models were trained on AEs that resulted from docetaxel treatment and would need to be evaluated against additional trials and additional treatments to determine if they are generalizable. A second limitation of this study is that there were no restrictions imposed on the number of clinical features that were used to develop prediction models. We chose to impose few restrictions on the model developers with the goal of determining whether the provided baseline clinical features could be used in any manner to build predictive models of treatment discontinuation. As a result, the models that were submitted to the challenge were not necessarily optimized for clinical translatability, but focused on addressing the proposed question.

The DREAM Challenge described here exemplifies how open clinical trial data can be used to explore new questions and highlights the role of open challenges as a tool for the development and objective evaluation of clinical models. We also demonstrated the willingness of the research community to work together. It should be noted that the group of researchers who performed the postchallenge analysis, developed the ensemble predictor, and wrote this manuscript had never worked together before. The challenges we face in biomedical science are too great for siloed research to be the status quo moving forward. Fostering research in this manner is additional evidence that the biomedical research of tomorrow can and will be a team effort.

## Appendix

### Prostate Cancer DREAM Challenge Community Members

**Kald Abdallah,** AstraZeneca, Gaithersburg, MD; **Antti Airola,** Department of Information Technology, University of Turku, Turku, Finland; **Tero Aittokallio,** Department of Mathematics and Statistics, University of Turku, Turku; Institute for Molecular Medicine Finland, University of Helsinki, Helsinki, Finland; **Catalina Anghel,** Informatics and Biocomputing Program, Ontario Institute for Cancer Research, Toronto, Canada; **Donna P. Ankerst,** Department of Mathematics, Technische Universität München, Munich, Germany; **Helia Azima,** Electrical and Computer Engineering Department, Ryerson University, Toronto, Canada; **Robert Baertsch,** Department of Biomolecular Engineering and Center for Biomolecular Science and Engineering, University of California, Santa Cruz, CA; **Pedro J. Ballester,** Cancer Research Centre of Marseille, Marseille, France; Sage Bionetworks, Seattle, WA; **Chris Bare,** Sage Bionetworks, Seattle, WA; **Vinayak Bhandari,** Ontario Institute for Cancer Research, Toronto, Canada; **Brian M. Bot,** Sage Bionetworks, Seattle, WA; **Ann-Sophie Buchardt,** University of Copenhagen, Copenhagen, Denmark; **Ljubomir Buturovic,** Clinical Persona, East Palo Alto, CA; **Da Cao,** University of Pennsylvania, Philadelphia, PA; **Prabhakar Chalise,** Department of Biostatistics, University of Kansas Medical Center, Kansas City, KS; **Billy H.W. Chang,** Division of Biostatistics, Jockey Club School of Public Health and Primary Care, The Chinese University of Hong Kong; **Junwoo Cho,** Department of Statistics, Kyungpook National University, Daegu, South Korea; **Tzu-Ming Chu,** JMP Life Sciences Division, SAS Institute, Cary, NC; **R. Yates Coley,** Department of Biostatistics, Johns Hopkins University, Baltimore, MD; **Sailesh Conjeti,** Computer Aided Medical Procedures, Technische Universität München, Munich, Germany; **Sara Correia,** Department of Informatics, Centre of Biological Engineering, University of Minho, Minho, Portugal; **James C. Costello,** Department of Pharmacology & Computational Biosciences Program, University of Colorado Comprehensive Cancer Center, University of Colorado, Anschutz Medical Campus, Aurora, CO; **Ziwei Dai,** Center for Quantitative Biology, Peking University, Beijing, China; **Junqiang Dai,** Department of Biostatistics, University of Kansas Medical Center, Kansas City, KS; **Cuong C. Dang,** Cancer Research Centre of Marseille, Marseille, France; Sage Bionetworks, Seattle, WA; **Philip Dargatz,** Johannes Wesling Klinikum Minden, Minden, Germany; **Sam Delavarkhan,** Electrical and Computer Engineering Department, Ryerson University, Toronto, Canada; **Detian Deng,** Department of Biostatistics, Johns Hopkins University, Baltimore, MD; **Ankur Dhanik,** Regeneron Pharmaceuticals, Tarrytown, NY; **Yu Du,** Department of Biostatistics, Johns Hopkins University, Baltimore, MD; University of Copenhagen, Copenhagen, Denmark; **Aparna Elangovan,** Computer Science Department, University of Melbourne, Melbourne, Australia; **Shellie Ellis,** Department of Health Policy and Management, University of Kansas Medical Center, Kansas City, KS; **Laura L. Elo,** Turku Centre for Biotechnology, University of Turku and Åbo Akademi University; Department of Mathematics and Statistics, University of Turku, Turku, Finland; **Shadrielle M. Espiritu, Fan Fan,** Ontario Institute for Cancer Research, Toronto, Canada; **Ashkan B. Farshi,** Electrical and Computer Engineering Department, Ryerson University, Toronto, Canada; **Ana Freitas,** Centre of Biological Engineering, University of Minho, Minho, Portugal; **Brooke Fridley,** Department of Biostatistics, University of Kansas Medical Center, Kansas City, KS; **Christiane Fuchs,** Institute of Computational Biology, Helmholtz Zentrum München; Department of Mathematics, Technische Universität München, Munich, Germany; **Eyal Gofer,** The Rachel and Selim Benin School of Computer Science and Engineering, The Hebrew University, Jerusalem, Israel; **Agnieszka K. Golińska,** Faculty of Mathematics and Informatics, University of Bialystok, Bialystok, Poland; **Stefan Graw,** Department of Biostatistics, University of Kansas Medical Center, Kansas City, KS; **Russ Greiner,** Department of Computing Science, University of Alberta; Alberta Innovates Centre for Machine Learning, Edmonton, Alberta, Canada; **Justin Guinney,** Sage Bionetworks, Seattle, WA; **Jing Guo,** Cancer Science Institute of Singapore, National University of Singapore, Singapore; Research and Development Department, Annoroad Gene Technology Co Ltd, Beijing, China; **Pankaj Gupta,** Computer Aided Medical Procedures, Technische Universität München, Munich, Germany; **Anna I. Guyer,** Department of Biology, Brigham Young University, Provo, UT; **Jiawei Han,** Department of Computer Science, The University of Illinois at Urbana-Champaign, IL; **Niels R. Hansen,** University of Copenhagen, Copenhagen, Denmark; **Outi Hirvonen,** Department of Oncology and Radiotherapy, Turku University Central Hospital, Turku, Finland; **Barbara Huang,** Ontario Institute for Cancer Research, Toronto, Canada; **Chao Huang,** Biostatistics and Imaging Analysis Lab, University of North Carolina at Chapel Hill, NC; **Jinseub Hwang,** Department of Computer Science and Statistics, Daegu University Daegu, South Korea; **Joseph G. Ibrahim,** Biostatistics and Imaging Analysis Lab, University of North Carolina at Chapel Hill, NC; **Vivek Jayaswal,** Biocon Bristol-Myers Squibb Research Centre, Bangalore, India; **Jouhyun Jeon,** Informatics and Biocomputing Program, Ontario Institute for Cancer Research, Toronto, Canada**; Zhicheng Ji,** Department of Biostatistics, Johns Hopkins University, Baltimore, MD; **Deekshith Juvvadi,** Jeevomics Pvt Ltd, New Delhi, India; **Sirkku Jyrkkiö,** Department of Oncology and Radiotherapy, Turku University Central Hospital, Turku, Finland; **Kimberly Kanigel-Winner,** University of Colorado, Anschutz Medical Campus, Aurora, CO; **Amin Katouzian,** Computer Aided Medical Procedures, Technische Universität München, Munich, Germany; **Marat D. Kazanov,** Research and Training Center on Bioinformatics, Institute for Information Transmission Problems, Russian Academy of Sciences, Moscow, Russia; **Suleiman A. Khan,** Institute for Molecular Medicine Finland, University of Helsinki, Helsinki, Finland; **Shahin Khayyer,** Electrical and Computer Engineering Department, Ryerson University, Toronto, Canada; **Dalho Kim,** Department of Statistics, Kyungpook National University, Daegu, South Korea; **Devin Koestler,** Department of Biostatistics, University of Kansas Medical Center, Kansas City, KS; **Fernanda Kokowicz,** Plant Morphogenesis and Biochemistry Laboratory, Federal University of Santa Catarina, Florianopolis, Brazil; **Ivan Kondofersky,** Norbert Krautenbacher, Institute of Computational Biology, Helmholtz Zentrum München; Department of Mathematics, Technische Universität München, Munich, Germany; **Damjan Krstajic,** Research Centre for Cheminformatics, Beograd, Serbia; Clinical Persona, East Palo Alto, CA; **Luke Kumar,** Department of Computing Science, University of Alberta, Edmonton, Alberta, Canada; **Christoph Kurz,** Institute of Health Economics and Health Care Management, Helmholtz Zentrum München, Munich, Germany; **Matthew Kyan,** Electrical Engineering and Computer Science Department, York University, Toronto, Canada; **Teemu D. Laajala,** Department of Mathematics and Statistics, University of Turku, Turku; Institute for Molecular Medicine Finland, University of Helsinki, Helsinki, Finland; **Michael Laimighofer,** Institute of Computational Biology, Helmholtz Zentrum München; Department of Mathematics, Technische Universität München, Munich, Germany; **Eunjee Lee,** Biostatistics and Imaging Analysis Lab, University of North Carolina at Chapel Hill, NC; **Wojciech Lesiński,** Faculty of Mathematics and Informatics, University of Bialystok, Bialystok, Poland; **Miaozhu Li,** Biodemography of Aging Research Unit, Center for Population Health and Aging, Social Science Research Institute, Duke University, Durham, NC; **Ye Li,** School of Computer Science, Shanghai Key Lab of Data Science, Fudan University, Shanghai, China; **Qiuyu Lian,** Tsinghua University, Beijing, China; **Xiaotao Liang,** School of Computer Science, Shanghai Key Lab of Intelligent Information Processing, Fudan University, Shanghai, China; **Minseong Lim,** Department of Statistics, Kyungpook National University, Daegu, South Korea; **Henry Lin,** Department of Computer Science, The University of Illinois at Urbana-Champaign, IL; **Xihui Lin,** Informatics and Biocomputing Program, Ontario Institute for Cancer Research, Toronto, Canada; **Jing Lu,** Department of Computational Medicine and Bioinformatics, University of Michigan, Ann Arbor, MI; **Mehrad Mahmoudian,** Turku Centre for Biotechnology, University of Turku and Åbo Akademi University, Turku, Finland; **Roozbeh Manshaei,** Electrical and Computer Engineering Department, Ryerson University, Toronto, Canada; **Richard Meier,** Department of Biostatistics, University of Kansas Medical Center, Kansas City, KS; **Dejan Miljkovic,** Computer Aided Medical Procedures, Technische Universität München, Munich, Germany; **Tuomas Mirtti,** Institute for Molecular Medicine Finland, University of Helsinki; Department of Pathology, Helsinki University Hospital, Helsinki, Finland; **Krzysztof Mnich,** Computational Centre, University of Bialystok, Bialystok, Poland; **Nassir Navab,** Computer Aided Medical Procedures, Technische Universität München, Munich, Germany; **Elias C. Neto,** Sage Bionetworks, Seattle, WA; **Yulia Newton,** Department of Biomolecular Engineering and Center for Biomolecular Science and Engineering, University of California, Santa Cruz, CA; **Thea Norman,** Sage Bionetworks, Seattle, WA; **Tapio Pahikkala,** Department of Information Technology, University of Turku, Finland; **Subhabrata Pal,** Centre for Cellular and Molecular Platforms, Bangalore, India; **Byeongju Park,** Department of Statistics, Kyungpook National University, Daegu, South Korea; **Jaykumar Patel,** Department of Computing Science, University of Alberta, Edmonton, Canada; **Swetabh Pathak,** Jeevomics Pvt Ltd, New Delhi, India; **Alejandrin Pattin,** Computer Aided Medical Procedures, Technische Universität München, Munich, Germany; **Gopal Peddinti,** Institute for Molecular Medicine Finland, University of Helsinki, Finland; **Jian Peng,** Department of Computer Science, The University of Illinois at Urbana-Champaign, IL; **Anne H. Petersen,** University of Copenhagen, Copenhagen, Denmark; **Robin Philip,** Jeevomics Pvt Ltd, New Delhi, India; **Stephen R. Piccolo,** Department of Biology, Brigham Young University, Provo, UT; **Sebastian Pölsterl,** Computer Aided Medical Procedures, Technische Universität München, Munich, Germany; **Aneta Polewko-Klim,** Faculty of Mathematics and Informatics, University of Bialystok, Bialystok, Poland; **Karthik Rao,** School of Medicine, Johns Hopkins University, Baltimore, MD; **Xiang Ren,** Department of Computer Science, The University of Illinois at Urbana-Champaign, IL; **Miguel Rocha,** Department of Informatics, Centre of Biological Engineering, University of Minho, Minho, Portugal; **Witold R. Rudnicki,** Faculty of Mathematics and Informatics, Computational Centre, University of Bialystok, Bialystok; Interdisciplinary Centre for Mathematical and Computational Modelling, University of Warsaw, Warsaw, Poland; **Charles J. Ryan,** Genitourinary Medical Oncology Program, Division of Hematology & Oncology, University of California, San Francisco, CA; **Hyunnam Ryu,** Department of Statistics, Kyungpook National University, Daegu, South Korea; **Oliver Sartor,** Tulane Cancer Center, Tulane University, New Orleans, LA; **Hagen Scherb,** Institute of Computational Biology, Helmholtz Zentrum München, Munich, Germany; **Raghav Sehgal,** Jeevomics Pvt Ltd, New Delhi, India; **Fatemeh Seyednasrollah,** Turku Centre for Biotechnology, University of Turku and Åbo Akademi University; Department of Mathematics and Statistics, University of Turku, Turku, Finland; **Jingbo Shang,** Department of Computer Science, The University of Illinois at Urbana-Champaign, IL; **Bin Shao,** Center for Quantitative Biology, Peking University, Beijing, China; **Liji Shen,** Sanofi, Bridgewater, NJ; **Howard Sher,** Sidney Kimmel Center for Prostate and Urologic Cancers, Memorial Sloan-Kettering Cancer Center and Weill Cornell Medical College, New York, NY; **Motoki Shiga,** Department of Electrical, Electronic and Computer Engineering, Gifu University, Gifu, Japan; **Artem Sokolov,** Department of Biomolecular Engineering and Center for Biomolecular Science and Engineering, University of California, Santa Cruz, CA; **Julia F. Söllner,** Institute of Computational Biology, Helmholtz Zentrum München, Munich, Germany; **Lei Song,** National Cancer Institute, National Institutes of Health, Rockville, MD; **Howard Soule,** Prostate Cancer Foundation, Santa Monica, CA; **Gustavo Stolovitzky,** IBM T.J. Watson Research Center, IBM, Yorktown Heights, NY; **Josh Stuart,** Department of Biomolecular Engineering and Center for Biomolecular Science and Engineering, University of California, Santa Cruz, CA; **Ren Sun,** Informatics and Biocomputing Program, Ontario Institute for Cancer Research; Department of Pharmacology and Toxicology, University of Toronto, Toronto, Canada; **Christopher J. Sweeney,** Department of Medical Oncology, Dana-Farber Cancer Institute and Brigham and Women’s Hospital, Harvard Medical School, Boston, MA; **Nazanin Tahmasebi,** Department of Computing Science, University of Alberta, Edmonton, Canada; **Kar-Tong Tan,** Cancer Science Institute of Singapore, National University of Singapore, Singapore; **Lisbeth Tomaziu,** University of Copenhagen, Copenhagen, Denmark; **Joseph Usset,** Department of Biostatistics, University of Kansas Medical Center, Kansas City, KS; **Yeeleng S. Vang,** Department of Computer Science, University of California Irvine, Irvine, CA; **Roberto Vega,** Department of Computing Science, University of Alberta, Edmonton, Canada; **Vitor Vieira,** Centre of Biological Engineering, University of Minho, Minho, Portugal; **David Wang,** Ontario Institute for Cancer Research, Toronto, Canada; **Difei Wang,** Department of Biochemistry and Molecular and Cellular Biology, Georgetown University Medical Center, Washington, DC; **Junmei Wang,** University of Texas Southwestern, Dallas, TX; **Lichao Wang,** Computer Aided Medical Procedures, Technische Universität München, Munich, Germany; **Sheng Wang,** Department of Computer Science, The University of Illinois at Urbana-Champaign, IL; **Tao Wang,** Quantitative Biomedical Research Center, Department of Clinical Sciences; Center for the Genetics of Host Defense, University of Texas Southwestern Medical Center, Dallas, TX; **Yue Wang,** Biostatistics and Imaging Analysis Lab, University of North Carolina at Chapel Hill, NC; **Russ Wolfinger,** JMP Life Sciences Division, SAS Institute, Cary, NC; **Chris Wong,** Department of Biomolecular Engineering and Center for Biomolecular Science and Engineering, University of California, Santa Cruz, CA; **Zhenke Wu,** Department of Biostatistics, Johns Hopkins University, Baltimore, MD; **Jinfeng Xiao,** Center for Biophysics and Quantitative Biology, The University of Illinois at Urbana-Champaign, IL; **Xiaohui Xie,** Department of Computer Science, University of California Irvine, Irvine, CA; **Doris Xin,** Department of Computer Science, The University of Illinois at Urbana-Champaign, IL; **Hojin Yang,** Biostatistics and Imaging Analysis Lab, University of North Carolina at Chapel Hill, NC; **Nancy Yu,** Informatics and Biocomputing Program, Ontario Institute for Cancer Research, Toronto, Canada; **Thomas Yu,** Sage Bionetworks, Seattle, WA; **Xiang Yu,** University of Pennsylvania, Philadelphia, PA; **Sulmaz Zahedi,** The Institute of Biomaterials and Biomedical Engineering, University of Toronto; iBEST-Li Ka Shing Institute of Knowledge, St Michael’s Hospital, Toronto, Canada; **Massimiliano Zanin,** INNAXIS Foundation & Research Institute, Madrid, Spain; **Chihao Zhang,** National Center for Mathematics and Interdisciplinary Sciences, Academy of Mathematics and Systems Science, Chinese Academy of Sciences, Beijing, China; **Jingwen Zhang,** Biostatistics and Imaging Analysis Lab, University of North Carolina at Chapel Hill, NC; **Shihua Zhang,** National Center for Mathematics and Interdisciplinary Sciences, Academy of Mathematics and Systems Science, Chinese Academy of Sciences, Beijing, China; **Yanchun Zhang,** School of Computer Science, Shanghai Key Lab of Data Science, Fudan University, Shanghai, China; **Fang Liz Zhou,** Sanofi, Bridgewater, NJ; **Hongtu Zhu,** Biostatistics and Imaging Analysis Lab, University of North Carolina at Chapel Hill, NC; **Shanfeng Zhu,** School of Computer Science, Shanghai Key Lab of Intelligent Information Processing, Centre for Computational Systems Biology, Fudan University, Shanghai, China; and **Yuxin Zhu,** Department of Biostatistics, Johns Hopkins University, Baltimore, MD.
